# The Role of mTORC1 Pathway and Autophagy in Resistance to Platinum-Based Chemotherapeutics

**DOI:** 10.3390/ijms241310651

**Published:** 2023-06-26

**Authors:** Zhenrui Pan, Hanxiao Zhang, Svetlana Dokudovskaya

**Affiliations:** CNRS UMR9018, Institut Gustave Roussy, Université Paris-Saclay, 94805 Villejuif, France; zhenrui.pan@gustaveroussy.fr (Z.P.); hanxiao.zhang@gustaveroussy.fr (H.Z.)

**Keywords:** mTORC1 pathway, autophagy, cisplatin, anticancer drug resistance

## Abstract

Cisplatin (*cis*-diamminedichloroplatinum I) is a platinum-based drug, the mainstay of anticancer treatment for numerous solid tumors. Since its approval by the FDA in 1978, the drug has continued to be used for the treatment of half of epithelial cancers. However, resistance to cisplatin represents a major obstacle during anticancer therapy. Here, we review recent findings on how the mTORC1 pathway and autophagy can influence cisplatin sensitivity and resistance and how these data can be applicable for the development of new therapeutic strategies.

## 1. Biochemical Mechanisms of Cisplatin Cytotoxicity

**Figure 1:**
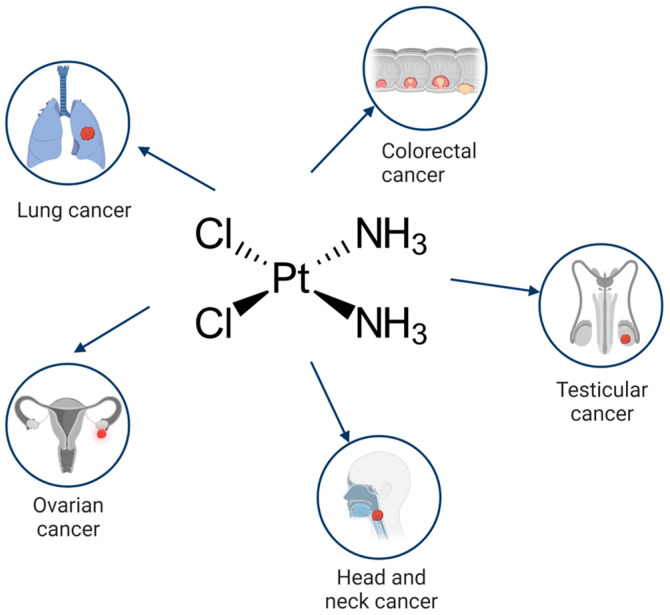
Cancers where cisplatin is used as a mainstream drug.

Cisplatin is a first line drug for many cancers, especially for lung, ovarian, head and neck, testicular and colorectal cancers (Figure 1). During anticancer therapy, cisplatin is injected intravenously. A high concentration of chloride ions (~100 mM) in the bloodstream suppresses the hydrolysis of the drug and maintains it in a neutral state. The binding of cisplatin to the plasma proteins, mainly to albumin, results in the deactivation of up to 95% of the injected drug.

**Figure 2:**
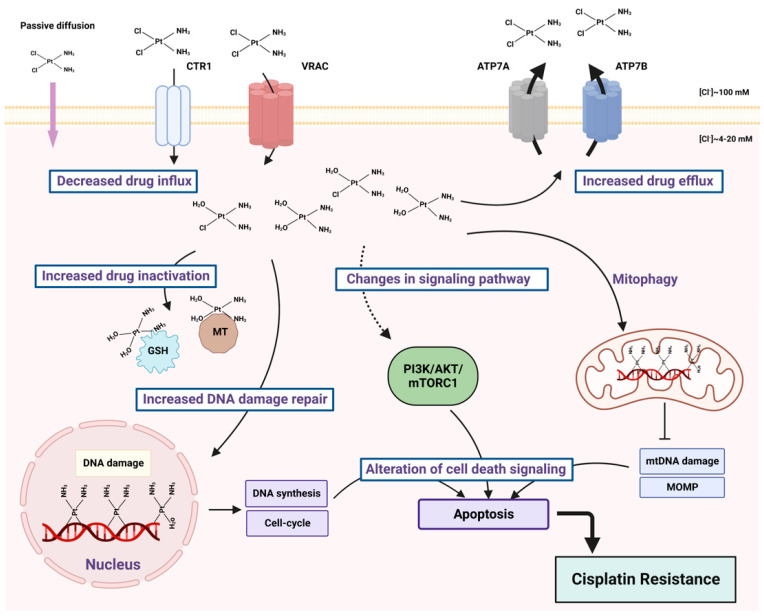
Main factors of cisplatin resistance. Cancer cells can develop resistance to cisplatin via decreased drug influx or increased efflux, because of the drug inactivation through the interaction with glutathione (GSH) and metallothioneins (MT), because of enhanced DNA damage response and alternations in signaling pathways.

Cisplatin enters the cells by passive diffusion through plasma membrane or by active transport via copper influx transporter 1 (CTR1) [1] and volume-regulated anion channel (VRAC) [2] (Figure 2). The majority of cytoplasmic cisplatin accumulates in vesicles that contain copper efflux transporters ATP7A or ATP7B rather than being diffusely localized throughout the cytoplasm. These intracellular secretory vesicles can further traffic and fuse with the plasma membrane, releasing cisplatin by exocytosis as a free drug, as a conjugate or as a complex with cellular proteins [3]. Copper transporters can be used by malignant cells to detoxify cisplatin, thereby promoting tumor resistance to chemotherapy [4].

Once in the cytoplasm, cisplatin undergoes aquation due to much lower chloride concentrations (~4–20 mM) than in the bloodstream. The displacement of one or two chloride ions by water molecules results in the generation of a highly electrophilic molecule that can interact with nucleic acids, phospholipids and proteins.

DNA is a primary target of cisplatin. Aquated cisplatin induces DNA damage by the forming of intrastrand and interstrand DNA cross-links through the preferential binding to the N^7^ position of guanine [5]. DNA lesions that are not resolved by DNA repair pathways block the production of DNA, mRNA and proteins and activate several transduction pathways, which finally lead to necrosis or apoptosis. Many cancers have defective DNA repair pathways; therefore, while normal cells can cope with the harm caused by cisplatin, cancer cells will die.

It is estimated that only ~1% of cisplatin interacts with nuclear DNA [6], the rest interacts with the mitochondrial DNA (mtDNA), lipids and sulfur donors (thiols as well as cysteines, methionines of cytoplasmic, mitochondrial and membrane proteins) [7,8,9]. The binding of aquated cisplatin to cytoplasmic molecules, including reduced glutathione (GSH) and metallothioneins (MTs), results in the accumulation of reactive oxygen species (ROS) that also triggers mitochondrial outer membrane permeabilization (MOMP) and DNA damage [8]. Cisplatin forms 4–6 times more DNA adducts with mtDNA than with gDNA. One of the reasons for the preference for mtDNA is due to a higher initial binding, because of the absence of histones in mitochondria. Another important reason is that nucleotide excision repair (NER), a major pathway for removing cisplatin damage in genomic DNA, does not function in mitochondria [10], which leads to the accumulation of cisplatin–DNA adducts. Increased mitochondrial biogenesis and dynamic alteration are essential factors in developing drug resistance [8,11].

**Figure 3:**
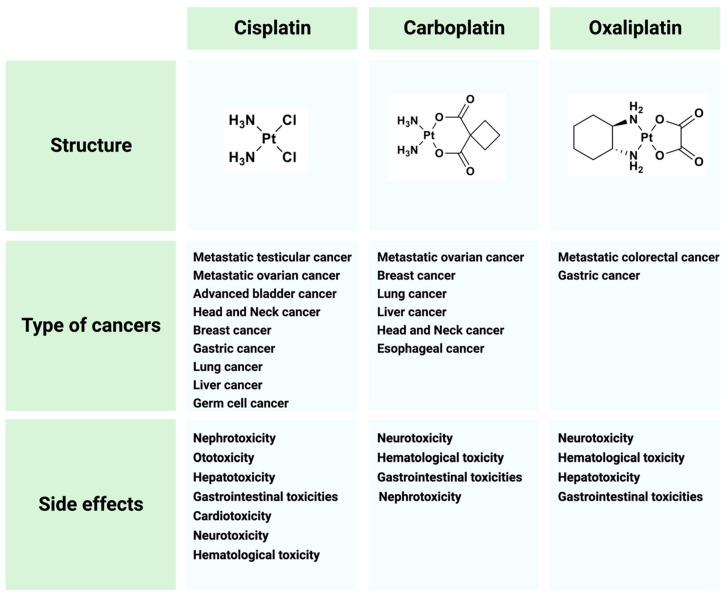
Cisplatin and its derivatives used in the treatment of different types of cancers. Side effects for each drug are listed.

Cisplatin does not show its highest potential in anticancer treatment because of side effects and drug resistance. The major adverse events that arise from cisplatin therapy are nephro-, hepato-, neuro- and gastrointestinal toxicities [12,13]. To overcome these limitations, great efforts have been made to search for cisplatin analogues which are better tolerated by patients and/or show anticancer activity in cisplatin-resistant tumors. The most widely used cisplatin derivatives are carboplatin and oxaliplatin (Figure 3). Carboplatin shows less toxicity than cisplatin at equimolar concentrations. It is used as a first-line treatment for patients with advanced ovarian cancer and advanced small-cell and non-small-cell lung cancer, while it shows a lower activity than cisplatin in germ cell tumors, bladder and head and neck cancers [14]. While the adducts formed by cisplatin and carboplatin are identical, the rate of formation of adducts is 10 times slower and 20- to 40- times higher concentrations of carboplatin are needed to obtain the same number of adducts [15]. Oxaliplatin forms different types of adducts than cisplatin and carboplatin, resulting in an altered DNA damage response. Oxaliplatin is used in combination with 5-fluorouracil for the treatment of metastatic colorectal cancer, which is insensitive to treatment with cisplatin and carboplatin [16].

Drug resistance is a serious problem in the treatment with platinum-based drugs. Cisplatin resistance could be both intrinsic (occurs from the beginning of treatment) and acquired (initially sensitive cells develop resistance to the drug over time). One of the major determinants of resistance is the type of cancer [17]. For example, small-cell lung cancer (SCLC; 10–15% of all lung cancers) is very sensitive to cisplatin, while non-small-cell lung cancer (NSCLC; about 80% to 85% of lung cancers) is quite resistant. Many cancers will eventually develop resistance against the drug with one notable exception, testicular germ cell cancer, which demonstrates a durable complete remission of ~80% of the patients treated with cisplatin.

Resistance to cisplatin depends on both the inner adaptive mechanisms of cancer cells and the tumor microenvironment, where hypoxic conditions increase the tolerance of cancer cells to the drug [18,19] (Figure 2). Among intercellular adaptive factors, the most important are: (1) a reduced drug accumulation due to either a decreased influx or an increased efflux; (2) an increase in DNA repair and changes in DNA damage response (DDR); (3) an alteration of apoptosis; (4) changes in signaling pathways, notably the mTORC1 pathway. These factors were conveniently classified by Galluzi et al. as pretarget, on-target, post-target and off-target, respectively [20]. There are numerous reviews addressing pre-, on- and post-target mechanisms [20,21,22,23,24], although relatively few are devoted to the changes in signaling pathways [25,26,27]. Here, we describe recent findings that connect platinum-based resistance mechanisms with major metabolic signaling networks—the mTORC1 pathway and autophagy. Metabolic remodeling is one of the main characteristics of tumors. Changes in glucose, amino acid and lipid metabolism, all these processes being under the control of the mTORC1 pathway, affect cisplatin resistance in tumor cells [28].

## 2. Molecular Basis of the mTORC1 Pathway and Autophagy

Mammalian target of rapamycin (mTOR) is a serine/threonine protein kinase that belongs to the phosphatidylinositol 3-kinase PI3K-related family (PIKK). As a part of two structurally and functionally different complexes, mTOR complex 1 (mTORC1) and 2 (mTORC2), mTOR maintains the balance between anabolism and catabolism in response to nutritional or environmental conditions via the phosphorylation of its multiple substrates [29]. Among nearly 60 direct targets of mTORC1, the most known and well-characterized are p70S6 Kinase 1 (S6K1) and eukaryotic translation initiation factor 4E-binding protein 1 (4E-BP1), involved in protein translation; transcription factor EB (TFEB), important for lysosome biogenesis and lipid metabolism; and Unc-51-like autophagy activating kinase 1 (ULK1), a member of the autophagy initiation complex.

**Figure 4:**
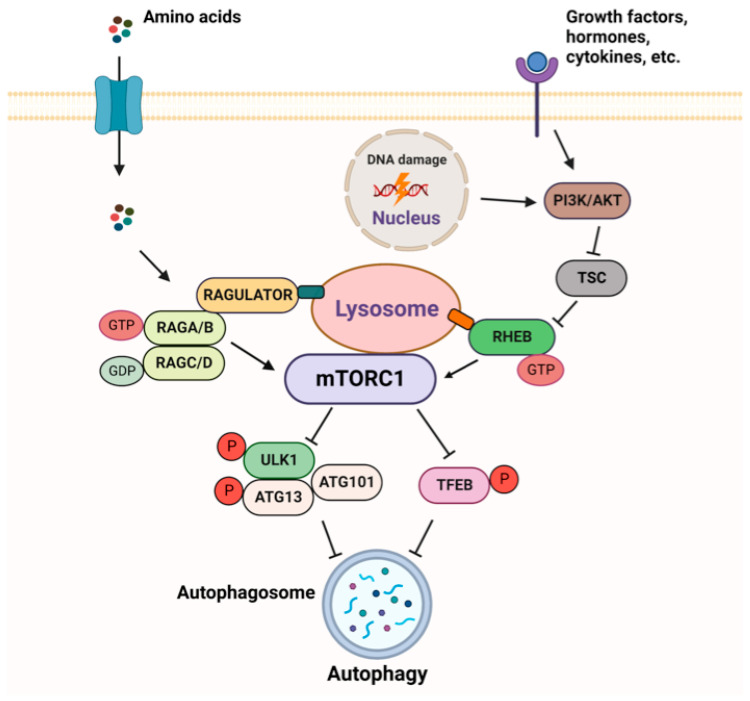
Overview of mTORC1 signaling.

The mTORC1 pathway is subjected to a tight regulation, allowing its activation, when growth factors, energy, and nutrients are sufficient [30,31] (Figure 4). In order to have an adequate and timely response to extra and intracellular inputs, mTORC1 responds to upstream signals through two different sets of the small GTPases–RHEB (Ras homologue enriched in brain) and RAGs (RAG guanosine triphosphatases). The activity of these GTPases depends on their effectors, GTPase-activating proteins (GAPs), which stimulate GTP hydrolysis, and guanine nucleotide exchange factors (GEFs).

The major site of mTORC1 activation is the lysosomal surface, where mTORC1 is recruited and induced in a RAG-GTPase-dependent manner when amino acids are abundant [32,33]. Four RAGs exist as heterodimers (i.e., RAGA (or RAGB) with RAGC (or RAGD)). In the presence of amino acids, RAGA/B is loaded with GTP while RAGC/D is bound to GDP. RAGs interact with the pentameric RAGULATOR complex, which exerts a GEF activity towards RAGA or RAGB [34,35]. Active RAGULATOR-RAG stimulates the recruitment of mTORC1 to the lysosomal membrane where it is fully activated by RHEB, loaded with GTP [36]. RHEB is under the control of another signaling node, the tuberous sclerosis (TSC) complex, which acts as a GAP to inhibit RHEB. TSC is a nexus of multiple physiological stimuli (e.g., energy status, growth factors, DNA damage) that signal to mTORC1 through the PI3K-AKT network [37]. mTORC1 is a well-recognized downstream effector of the PI3K/AKT signaling pathway, which responds to three main inputs: growth factors, hormones, chemokines and cytokines. RAG GTPases regulate the recruitment of TSC to the lysosome and its ability to interact with and inhibit RHEB in response to amino acid starvation, growth factors removal and to other stresses that inhibit mTORC1 [38,39,40]. Both RAGs and RHEB are necessary for mTORC1 activation at the lysosome [40]. When amino acids are scarce, GEFs and GAPs (e.g., GAP activity toward RAGs complex 1 (GATOR1) [41]) transform RAGs in their inactive form, which further leads to mTORC1 suppression.

mTORC1 plays a central role as a negative regulator of autophagy, a catabolic process by which cytosol and organelles are sequestered within double-membrane-bound vesicles that deliver their contents to the lysosome for degradation and recycling [42]. mTORC1 is active and autophagy is suppressed under optimal growth conditions, e.g., a sufficient quantity of amino acids and glucose. In order to inhibit autophagy initiation, active mTORC1 associates with a ULK1 protein complex and phosphorylates its members, ULK1 and autophagy-related protein 13 (ATG13) [43]. This phosphorylation prevents the kinase activity of the ULK1 complex. mTORC1 also regulates autophagy at the transcriptional level by modulating the localization of TFEB, which regulates the expression of autophagy and lysosomal genes. mTORC1 phosphorylates TFEB, thereby inhibiting TFEB activity when nutrients are present [44].

Finally, mTORC1 functioning is also important during DNA damage. The interaction between the DNA damage response and the mTORC1 pathway is especially relevant in the context of cisplatin treatment and can be considered as an outstanding example of how genotoxic stress and metabolic pathways can be triggered to overcome the disease [45]. Ideally, during anticancer therapy, the DDR and mTORC1 pathways should be repressed in cancer cells to provide a prodeath scenario, while in the healthy cells, these pathways should be active to repair damage and maintain proliferation to enable survival. Accordingly, one of the main causes of cancer therapy resistance is sustained DNA repair associated with hyperactivated mTORC1 signaling.

## 3. mTORC1 Pathway and Cisplatin Resistance

In normal physiological conditions, the PI3K/AKT/mTOR pathway undergoes stringent regulation to ensure the proper activity and balance necessary for healthy homeostasis. In the context of numerous cancers, this pathway exhibits a persistent state of activation. Thus, the aberrant activation of the pathway has been detected in ~70% of ovarian and breast cancers [46,47] and in ~90% of head and neck cancers and lung adenocarcinoma [26,48]. The mechanisms behind this activation include the amplification of the mutations of genes encoding PI3K subunits, AKT, inactivating mutations in TSC genes, or, conversely, activating mutations in MTOR [49]. Interestingly, the mutation frequency of the MTOR gene itself depends on the cancer type and is generally quite low (around 2–5%), especially in comparison with p53, which is mutated in more than 25% of cancers [50,51,52]. Cancer cells with a subset of mTOR-activating mutations are hypersensitive to the mTOR inhibitor rapamycin [51].

**Figure 5:**
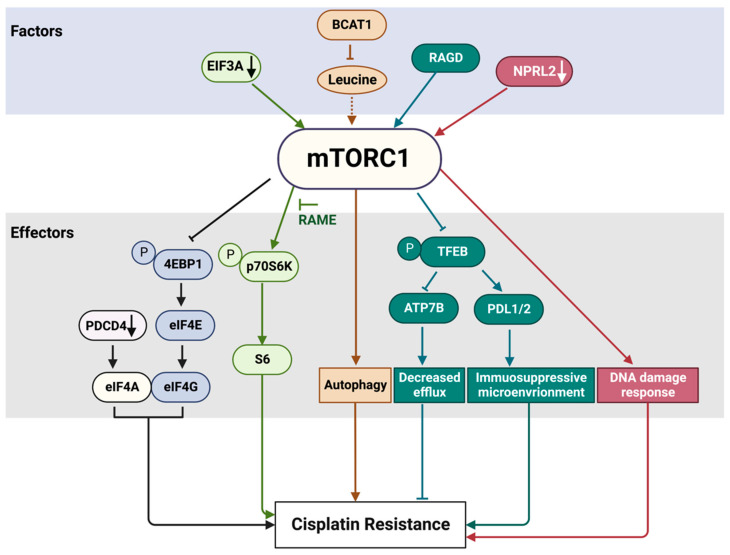
mTORC1 pathway factors and effectors involved in cisplatin resistance. The factors and effectors of the same signaling path are indicated with the same color. EIF3A translational factor negatively regulates mTORC1 activity. Accordingly, EIF3A downregulation (black arrow) increases mTORC1 activity. The activation of p70S6K by mTORC1 can be suppressed by RAME. Many oncogenic mutations have been detected in eukaryotic translation initiation factors, a group of mTORC1 downstream effectors. In addition, PDCD4, which is not a direct target of mTORC1, can suppress protein translation by interacting with EIF4A and EIF4G to inhibit the formation of the translation initiation complex. A knockdown of PDCD4 (black arrow) reduces sensitivity to platinum drugs. BCAT1 overexpression results in a decrease of leucine and other branched-chain amino acid levels. As a consequence, mTORC1 cannot be effectively activated (dashed arrow), resulting in enhanced autophagy and cisplatin resistance. NPRL2 downregulation (white arrow) results in mTORC1 activation, compromised DNA damage response and cisplatin resistance. See text for more details. RAGD promotes TFEB inhibition through its phosphorylation by mTORC1 and sequestration in the cytoplasm, where TFEB cannot exert its function as a transcription factor. Cisplatin treatment induces TFEB nuclear translocation and activation. Active TFEB increases the expression of programmed cell death-ligands 1 and 2 (PD-L1 and PD-L2) to foster an immunosuppressive tumor microenvironment that mediates drug resistance. The suppression of TFEB inhibits the expression of the copper transporter ATP7B involved in cisplatin efflux and sensitizes initially resistant ovarian cancer cells to cisplatin.

Tumors with acquired resistance to cisplatin often have a constitutive activation of mTORC1 signaling [53]. mTORC1 can influence cisplatin resistance in many ways—at the transcriptional and translational levels and by responding to various cues, such as amino acids, energy, and DNA damage (Figure 5). One of the most important functions of mTORC1 in cisplatin resistance takes place at the level of translational regulation. mTORC1 phosphorylation results in the activation of the p70S6K kinase activity, which, in turn, has many substrates, including ribosomal protein S6 [54]. In its unphosphorylated state, 4E-BP1 binds and inhibits eIF4E to prevent translation initiation. The phosphorylation of 4E-BP1 by mTORC1 releases eIF4E, which can now bind to eIF4G and initiate translation [55,56]. The phosphorylation of p70S6K and 4E-BP1 was elevated in cells that acquired resistance to cisplatin compared to parental sensitive cells in small-cell lung cancer [57] and ovarian cancer cells [58]. The S6K1 inhibitor RAME, which blocks the interaction between S6K1 and mTOR, promoted autophagy and apoptosis and greatly enhanced the antitumor effect of cisplatin in cisplatin-resistant cervical cancer cells [59].

Programmed cell death 4 (PDCD4) protein, which is not a direct target of mTORC1, can suppress protein translation by interacting with EIF4A and EIF4G to inhibit the formation of the translation initiation complex. An overexpression of *PDCD4* enhances platinum sensitivity, while a knockdown of PDCD4 reduces platinum sensitivity in ovarian cells and in a xenograft model [60].

EIF3A, the largest subunit of the eIF3 translational initiation complex, downregulates the translation of a number of nucleotide excision repair proteins. EIF3A knockdown or ectopic overexpression, respectively, increases or decreases cellular resistance to cisplatin in a number of cancer cell lines, including nasopharyngeal and ovarian carcinoma and lung cancer cell lines, likely due to EIF3A’s role in the regulation of NER proteins [61,62]. Another recently proposed mechanism is that EIF3A regulates S6K1 activity by inhibiting mTORC1 kinase via regulating the synthesis of its subunit RAPTOR. Thus, not only does mTORC1 regulate translational control, but it can also be regulated by translational control in a feedback loop, which involves S6K1, contributing to cellular response to cisplatin [63].

Translationally controlled tumor protein (TCTP) stimulates mTORC1 by positively regulating RHEB activity [64]. TCTP is overexpressed in many human tumor tissues [65]. The inhibition of mTORC1 by rapamycin in human lung cancer cells and an A549 lung cancer xenograft model induces ubiquitin–proteasome degradation of TCTP. Moreover, the minimal dose of rapamycin required to induce TCTP proteolysis enhances the efficacy of cisplatin through the induction of apoptotic cell death in vitro and in vivo. This synergistic cytotoxicity was induced irrespective of the functional status of p53 [66].

Chemotherapy can induce oxidative and genotoxic stress, triggering a senescence-like state, which in many cancer cells causes treatment resistance, supporting tumor proliferation and cancer recurrence. mTORC1 activity is upregulated in senescent cells, which are insensitive to serum and amino acid starvation. How this can be related to the senescence-induced drug resistance was addressed in a recent study by Jiang et al., who investigated the role of five small GTPases that can activate mTORC1 in response to amino acid stimulation [67]. In the senescence-like hepatoma cell line HepG2, RAGC and RHEB, but not RAB1A, RAB5 or ARF1, were required for persistent mTORC1 activity. One of the reasons could be that senescence-like cells rely on amino acid supply resulting from the lysosomal degradation of cellular components via autophagy. Out of the five studied small GTPases, only RAGC and RHEB localized to the lysosome, while RAB1A and ARF1 preferentially localized to ER/Golgi, and RAB5 to endosomes. The knocking down of RAGC or RHEB, but not the other three GTPases, increased cisplatin sensitivity in senescence-like HEPG2, which were significantly more resistant than their proliferating counterparts. Accordingly, the expression of RAGC and RHEB, but not the other GTPases, is associated with a poor prognosis in liver cancer patients. It will be important to find out if this effect can be observed in different tumors and if other RAGs can also have a role in the cisplatin resistance of senescence-like cancer cells. That would be of a particular interest, given the recent finding that RAGs in heterodimers are not functionally redundant. This is especially relevant to the function of transcription factors TFE3 and TFEB, which regulate the transcription of many lysosomal genes. mTORC1 phosphorylates TFE3 and TFEB at the lysosomal membrane, leading to their inactivation and sequestration in the cytoplasm [68]. RAGD preferentially promotes mTORC1 phosphorylation of TFEB, which is linked to a stronger association of RAGD with the lysosome, compared to RAGC, while TEF3 is preferentially recruited by RAGC [69]. Recent findings demonstrate that TFEB regulates the expression of ATP7B, a copper transporter involved in cisplatin efflux. The suppression of TFEB inhibits ATP7B expression and sensitizes initially resistant ovarian cancer cells to cisplatin [70]. In addition, in ovarian cancer cells, TFEB is involved in cisplatin resistance by regulating the tumor microenvironment. Cisplatin treatment induced TFEB nuclear translocation, increasing the expression of programmed cell death-ligands 1 and 2 (PD-L1 and PD-L2) to foster an immunosuppressive tumor microenvironment that mediates immune evasion and drug resistance [71].

An increased expression of branched-chain amino acid transaminase 1 (BCAT1) is correlated with a poor prognosis in many solid tumors and is also associated with decreased cisplatin sensitivity. BCAT1 is responsible for facilitating the synthesis of α-ketoglutarates from branched-chain amino acids (i.e., leucine), which are important activators of the mTORC1 pathway. A recent study has demonstrated that BCAT1 decreases cisplatin sensitivity by activating autophagy via the mTORC1 pathway [72]. Following treatment with cisplatin, BCAT1 expression is initially upregulated, resulting in a decrease in branched-chain amino acid levels due to the enzymatic activity of BCAT1. The deficiency of amino acids, especially leucine, suppresses mTORC1 activity and subsequently enhances autophagy, leading to a decrease in cisplatin sensitivity. Treatment with leucine or chloroquine, as well as the knockdown of BCAT1, inhibits autophagy and increases cisplatin sensitivity in cervical cancer and hepatocellular carcinoma cell lines and in mouse models. Thus, BCAT1 can be considered as a potential pharmacological target to overcome cisplatin resistance.

One of the major upstream regulators of the mTORC1 pathway, the GATOR1 complex, is involved in the regulation of nutrient sensing and responding. Various mutations of the genes encoding GATOR1 proteins have been detected in many solid tumors [73]. Notably, a low expression of NPRL2, one of the GATOR1 components, in different types of lung cancers is correlated with cisplatin resistance [74,75]. The overexpression of NPRL2 in NPRL2-deficient and cisplatin-resistant NSCLC cells reactivates cellular response to cisplatin and promotes tumor suppression activity in vitro and in mouse models [74]. The reason for this resistance is still not clear, but it could be related to the role of NPRL2 in DNA damage response [75,76].

Recently, a number of different mTOR inhibitors in combination with various platinum drugs have been applied to sensitize resistant cells. mTORC1 inhibitors can be generally divided into four groups: (1) rapamycin and its analogs (rapalogues), which preferentially target mTORC1, yet the 4E-BP1 branch is only partially sensitive to these inhibitors, activating a negative feedback loop of PI3K–AKT signaling; (2) pan-mTORC1 inhibitors (TORKIs), which target the catalytic core of mTOR kinase and therefore suppress both mTORC1 and mTORC2 (pp242, torin1, AZD8055); (3) bivalent inhibitors that target two domains of mTOR and are effective in inhibiting the 4E-BP1 phosphorylation (RapaLink1); and (4) dual inhibitors that can act on mTOR kinase and another protein, most often targeting PI3K/AKT signaling (NVP-BEZ235) [45].

The suppression of the mTOR activity by various inhibitors has been applied to enhance cisplatin-induced apoptosis in ovarian cancer [77], head and neck cancer [26], hepatocellular carcinoma [78], lung cancer [79] and nasopharyngeal carcinoma [80]. The treatment of platinum-resistant ovarian cancer cells with carboplatin and a dual mTORC1/2 inhibitor resulted in a strong inhibition of cancer cell proliferation, improved tumor control, reduced metastasis and significantly increased survival in mice [77]. Due to these promising results, several clinical trials (phase I/II) have been initiated recently in patients with triple-negative breast cancer to see the effects of a combined therapy involving platinum drugs and the mTORC1 inhibitor everolimus [81] or the dual PI3K/mTOR inhibitor gedatolisib [82]. The results of these clinical trials did not demonstrate a significantly improved response rate compared to cisplatin monotherapy. However, these results may have been compromised by the small sample size (14 patients in the first trial, ~100 patients in the second) and nonrandomized study design. Earlier clinical trials on different solid tumors (phase I only) had a very small patient cohort and did not provide conclusive results [83,84,85]. Further clinical trials encompassing a broader range of cancer types and larger patient cohorts are necessary to determine the efficacy of combining platinum drugs with PI3K/mTOR inhibitors and their potential to improve the outcomes of cancer patients.

## 4. Autophagy in Cisplatin Resistance

**Figure 6:**
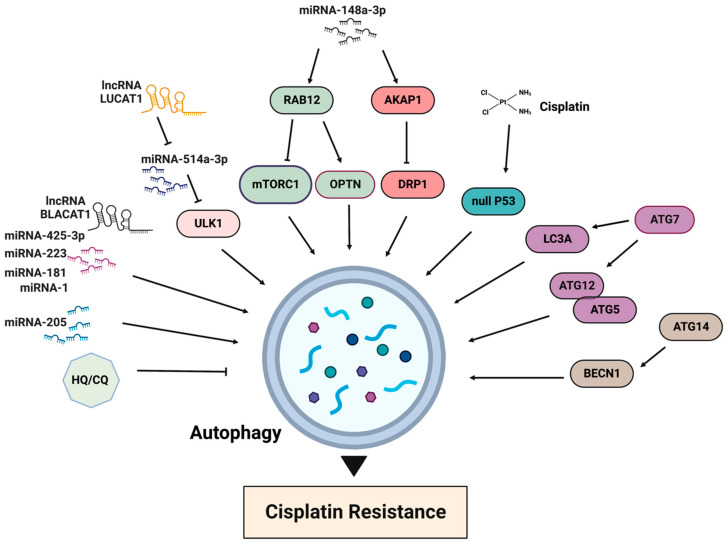
Main factors of autophagy involved in cisplatin resistance. HQ/CQ: chloroquine and hydroxychloroquine.

The activation of the mTORC1 pathway results in autophagy inhibition, while cisplatin treatment generally promotes autophagy (Figure 6). One of the first reports about the association between autophagy and cisplatin resistance dates back to 2010, when it was demonstrated that the acquired cisplatin resistance in lung adenocarcinoma cells A549 was associated with elevated autophagy [86]. The inhibition of autophagy is often observed in cisplatin-sensitive cells, whereas the basal level of autophagy is elevated in cisplatin-resistant cells. Accordingly, the suppression of autophagy, for example by chloroquine, increases drug toxicity and can improve sensitivity in cisplatin-resistant cancer cells [87]. A recently published resource database of genes associated with platinum resistance in cancer demonstrates that genes involved in the production of autophagosomes, including ATG5, ATG7, ATG12, ATG14 and BECN1, promote platinum resistance [88]. In the same line, an elevated expression of LC3A was shown to be associated with platinum resistance and a worse prognosis in ovarian clear cell carcinomas [89]. Thus, the inhibition of autophagy can be considered as a strategy for improving cisplatin sensitivity.

Autophagy can also have a cytoprotective function, which is particularly important in the context of adverse effects during cisplatin treatment. Notably, up to 30% of patients receiving cisplatin develop acute kidney injury (AKI), leading to a rapid loss of renal function or renal failure. Autophagy is activated in renal tubules to protect against neurotoxicity during the acute phase. On the other hand, the sustained activation of autophagy will limit kidney repair. Thus, it is important to manipulate autophagy differently at the beginning and after the cisplatin treatment, in order to protect kidneys and allow for their effective recovery [90]. A recent review on cisplatin and autophagy extensively considered many aspects of this catabolic process in drug treatment [91]. Therefore, here, we only outline the most important features of the role of autophagy in cisplatin resistance.

Following cisplatin treatment, autophagy induction is detected in both cisplatin-sensitive and resistant cancer cells. Therefore, drug resistance is not necessarily a consequence of autophagy induction. It is important, though, to evaluate what type of autophagy is induced upon drug treatment because autophagy can participate in both cell survival (cytoprotective autophagy) and cell death (cytotoxic autophagy) [92]. Moreover, Gewirtz proposed to consider two additional types of autophagy: nonprotective and cytostatic [93]. These four forms of autophagy can be identified by the functional outcomes of their inhibition by drugs, but so far, there are no reliable morphological or biochemical methods that would allow us to distinguish between autophagy types. Cisplatin generally induces autophagy, which has a prosurvival role for cancer cells and, therefore, can participate in drug resistance. The inhibition of this cytoprotective autophagy results in chemosensitization. In contrast, the inhibition of nonprotective autophagy does not lead to changes in drug sensitivity [94,95]. Moreover, the functions of autophagy can be changed in response to stimuli, stress or genetic alterations through a so-called “autophagic switch”.

One example of this switch relevant to cisplatin treatment was recently demonstrated by Patel et al. when comparing wild-type and functionally null p53 NSCLC H460 cells [94]. Cisplatin induced cytoprotective autophagy in p53-deficient cells, while p53 wild-type cells had nonprotective autophagy. p53 wild-type H460 cells, where autophagy was nonprotective, were more sensitive to cisplatin than the H460crp53 cells. However, both types of cells had the same degree of apoptosis. Thus, the type of autophagy, and not p53 function, largely determined cisplatin sensitivity. The inhibition of cytoprotective autophagy was sufficient to restore cisplatin sensitivity in p53-deficient cells [94]. Therefore, the combination of autophagy inhibitors and cisplatin seems to have a strong therapeutic potential for resistant tumors, where autophagy plays a cytoprotective role, but this may not be applicable to cisplatin-sensitive cancers. Nevertheless, it is currently unclear how these findings can be applied in the clinic, especially because there are no valid methods to identify the nature of induced autophagy.

Many reports describe the regulation of cisplatin-induced autophagy in tumor cells by various noncoding RNAs (ncRNAs), including long noncoding RNAs, microRNAs and circular RNAs [91,96]. In the majority of cases, these ncRNAs inhibit autophagy via different mechanisms and increase sensitivity to the drug in both sensitive and resistant cancer cells. For example, the inhibition of autophagy via miR-205 sensitized castration-resistant prostate cancer cells to cisplatin [97]. Another study demonstrated that the overexpression of miRNA-1, -181, -223, -425-3p and lncRNA BLACAT1 in cisplatin-resistant lung cancer cells upregulated cisplatin-induced autophagy [91]. LncRNA LUCAT1, highly expressed in cisplatin-resistant NSCLC tissues and cells, contributes to cisplatin resistance by negatively regulating miR-514a-3p, another noncoding RNA. miR-514a-3p, in turn, negatively modulates ULK1 expression. LUCAT1 silencing enhances cisplatin sensitivity by inducing cell apoptosis, suppressing autophagy and inhibiting cell metastasis in NSCLC. Moreover, LUCAT1 regulates the sensitivity of NSCLC cells to cisplatin by upregulating ULK1 via sponging miR-514a-3p [98].

The inhibition of autophagy via different micro-RNAs also sensitized gastric cancer cells to cisplatin [99,100]. Thus, miR-148a-3p modulates cisplatin sensitivity by simultaneously regulating RAB12-mediated autophagy and AKAP1-mediated mitochondrial fission [100]. RAB12 GTPase, a member of the RAS oncogene family, induces autophagy by inhibiting mTORC1 activity [101] and accelerating autolysosome maturation [102]. RAB12 also interacts with optineurin (OPTN), an important mitophagy receptor. The suppression of mTORC1 by RAB12 facilitates early autophagosome formation to protect gastric cancer cells from cisplatin-induced cell death. miR-148a-3p can significantly reduce autophagic flux and autophagosome formation by regulating RAB12. A-kinase anchoring protein 1 (AKAP1) is upregulated in cisplatin-resistant gastric cancer tissues and antagonizes cisplatin-induced mitochondrial fission by the phosphorylation of dynamin-related proteins 1 (DRP1), an important mitochondrial fission factor [100]. Because AKAP1 restrains mitochondrial fission and reinforces cisplatin resistance in gastric cancer cells, targeting this protein by miR-148a-3 sensitizes cells to cisplatin. The role of DRP1 in cisplatin resistance is somewhat controversial and can be the opposite in different cancers. For example, in cisplatin-resistant ovarian cancer cell lines SKOV3, DRP1 expression was downregulated, and the knockdown of DRP1 in parental sensitive cell lines provoked cisplatin resistance [11]. Similarly, the ROS-promoted downregulation of DRP1 phosphorylation triggered mitochondrial fission and cisplatin resistance in a number of ovarian cancer cells. The inhibition of DRP1 by the specific inhibitor Mdivi-1 increased the cisplatin sensitivity of ovarian cancer cells under hypoxia [103]. On the other hand, the inhibition of DRP1 by Mdivi-1 sensitized lung, breast, colon and renal carcinoma cell lines to cisplatin [104].

Despite promising in vivo and in vitro results in the application of noncoding RNAs to reverse drug resistance, these strategies are far from being applied in the clinic. The main difficulties concern the methods of expressing ncRNAs in a cancerous tissue or in organs, the dosage in combination with cisplatin and other anticancer drugs used in the patient undergoing therapy.

Finally, cisplatin resistance can also be associated with the changes of mitochondrial autophagy (mitophagy). As mentioned above, cisplatin activates DRP1, leading to mitochondrial fragmentation. This results in the segregation of dysfunctional organelles and their enhanced removal by mitophagy. The suppression of DRP1-mediated mitophagy by Mdivi-1 increases the sensitivity of hepatocellular carcinoma cells to cisplatin [105]. Similarly, in cisplatin-resistant ovarian carcinoma and osteosarcoma cell lines, mitochondria are more fragmented. These cells exhibit an increased expression of mitophagy receptor BNIP3. A silencing of BNIP3 resensitizes resistant cells to the drug [106]. A study in cisplatin-resistant lung cancer cells also revealed that under hypoxic conditions, BNIP3-dependent autophagy induction was enhanced, allowing cell survival, thus also pointing to the importance of the microenvironment in cisplatin resistance [107].

In contrast to mTORC1, which can now be targeted by the fourth generation of drugs, the only autophagy inhibitors on the market remain chloroquine and hydroxychloroquine. Recently, many efforts have been applied to search for autophagy-selective drugs, but none of them are currently found in clinical development [108]. However, given the progress in understanding the autophagy pathway at the molecular level that have been achieved during recent decades and a growing number of biotech companies investing in the developing of autophagic modulators, it is reasonable to expect the appearance of new drugs that can specifically inhibit (or induce) autophagy. Most probably, some of these drugs will be effective in combination with platinum drugs to sensitize cancers that have been resistant so far to chemotherapies.

## 5. Conclusions

Cisplatin resistance is a significant challenge in cancer treatment, leading to decreased efficacy and poorer patient outcomes. Recent studies on the involvement of the mTORC1 pathway and autophagy in this resistance mechanism offer potential avenues for therapeutic intervention. Many mTORC1 signaling components and effectors are frequently deregulated or altered in different cancers. Cisplatin-resistant tumors often have a constitutive activation of mTORC1 signaling. Targeting the mTORC1 pathway with inhibitors has shown promising results in restoring cisplatin sensitivity, sensitizing resistant cells to the drug. Autophagy has emerged as another important player in cisplatin resistance because it can have a prosurvival role by protecting cancer cells from cisplatin-induced stress. Combination therapies that inhibit mTORC1 while modulating autophagy may provide a more effective strategy to sensitize resistant cancer cells to cisplatin. More clinical trials covering different cancer types and larger patient cohorts are necessary to determine the efficacy of such therapies.

## Data Availability

Not applicable.
